# Determinants of postpartum depression among mothers in Debre Tabor town, North-central, Ethiopia: Community-based unmatched case-control study

**DOI:** 10.3389/fgwh.2022.910506

**Published:** 2022-10-12

**Authors:** Fentaw Teshome Dagnaw, Wondimnew Desalegn Addis, Desalegn Tesfa, Aragaw Tesfaw Desale, Nurayine Abubeker Issa, Yismaw Yimam Belachew, Getachew Yideg Yitbarek, Alebachew Taye Belay, Ermias Sisay Chanie, Habtamu Shimels Hailemeskel

**Affiliations:** ^1^Department of Public Health, College of Health Sciences, Debre Tabor University, Debre Tabor, Ethiopia; ^2^Department of Medicine, College of Health Sciences, Debre Tabor University, Debre Tabor, Ethiopia; ^3^Department of Biomedical Sciences, College of Health Sciences, Debre Tabor University, Debre Tabor, Ethiopia; ^4^Department of Statistics, College of Natural and Computational Sciences, Debre Tabor University, Debre Tabor, Ethiopia; ^5^Department of Pediatrics and Neonatal Nursing, College of Health Sciences, Debre Tabor University, Debre Tabor, Ethiopia

**Keywords:** postpartum depression, depressive disorder, postpartum mothers, Debre Tabor, Ethiopia

## Abstract

**Background:**

Postpartum depression (PPD) is a non-psychotic depressive disorder of variable severity, and it can begin as early as 2 weeks after delivery and can persist indefinitely if left untreated. In Ethiopia, the prevalence of postpartum depression is high. There is a dearth of literature to determine factors associated with postpartum depression in Ethiopia, specifically in the study area.

**Objective:**

This study aimed to identify factors associated with postpartum depression among mothers in Debre Tabor Town, Northcentral Ethiopia.

**Method:**

A community-based unmatched case-control study was conducted among mothers who were living in Debre Tabor Town and fulfilled the inclusion criteria. Postpartum mothers were selected using a simple random sampling technique from the listed sampling frame at the health center. Then, the sample cases and controls were interviewed until the sample size was fulfilled by using a consecutive sampling method. The data were entered into the EPI data version 4.6 and then imported and analyzed using SPSS version 25. Descriptive statistics of different variables were done by cross-tabulation. Binary logistic regression was used to assess the determinant factors with the outcome variable. A *P*-value of < 0.05 was considered to declare statistical significance.

**Results:**

A total of 308 postnatal mothers living in Debre Tabor Town were included, with a 97.5% response rate. History of substance use in the previous 3 months (AOR: 6.47, 95% CI; 2.61, 15.74), current baby illness (AOR: 3.9, 95% CI; 1.5, 10.12), marital dissatisfaction (AOR: 2.41, 95% CI; 1.22, 4.75), unplanned current pregnancy (AOR: 3.46, 95% CI; 1.32, 9.12), and breastfeeding (AOR: 0.22, 95% CI; 0.09, 0.55) were independent factors that affected the occurrence of PPD.

**Conclusion:**

This study revealed that a recent history of substance use (in the past 3 months), current baby illness, marital satisfaction, unplanned current pregnancy, and breastfeeding were associated with postpartum depression. Healthcare providers working in maternal and child health clinics and health extension workers should give special attention to postpartum mothers who have had a history of substance use, current baby illness, unplanned pregnancy, non-breastfeeding mothers, and mothers with poor marital satisfaction.

## Background

Postpartum depression (PDD) also known as postnatal depression is a non-psychotic depressive disorder of variable severity it can begin as early as 2 weeks after delivery and can persist indefinitely if left untreated. Depressive disorders are the leading cause of global disability, and PDD is a clinical depression with symptoms that can include a feeling of fatigue, social withdrawal, sadness, changes in sleeping and eating patterns, guilt, crying, loneliness, and low self-esteem lasting longer than 2 weeks (1–3). Nearly one-fifth of the global disease burden has been ascribed to neuropsychiatric disorders, including those disorders that occur during the postpartum period. These estimates indicate the importance of addressing mental disorders in public health (4). According to a standardized diagnostic and statistical manual, postpartum depression is one type of depressive episode, which occurs within 1 year of childbirth. However, it can also manifest as the occurrence of a major depressive episode within 4 weeks after delivery. The fundamental neurobiological changes result from developmental connections between genetic susceptibility and environmental factors rather than a simple chemical imbalance (1, 5).

In low and lower-middle-income countries, particularly among poorer women, the prevalence of postpartum depression is high (6, 7). The prevalence rates vary by geographic location, with Southern Africa having the highest rate (39.96%). In industrialized nations or high-income countries, there is a much lower prevalence of PPD. Furthermore, the findings from various studies show that when marital status, educational level, social support, spouse care, violence, gestational age, breastfeeding, child mortality, pregnancy plan, financial difficulties, partnership, life stress, smoking, alcohol intake, and living conditions were factored into the pooled estimates, there was a significant difference in PPD rates (8). In studies conducted in Ethiopia, the prevalence of postpartum depression is high in different places; for instance, in Bahir Dar, it is 22.2% (9), in Harar, 13.11% (10), in Debre Birhan, 15.6% (4), and Ankesha, 23.7% (11).

Maternal depression refers to a wide range of depressive disorders that can affect mothers (up to 12 months postpartum) and expectant moms. Prenatal depression, postpartum depression, and postpartum psychosis are examples of these depressive illnesses. Maternal depression is becoming more widely recognized as a global public health concern with far-reaching consequences for an individual's career, family, and the baby's health and development (12, 13). Untreated postpartum depression has a serious adverse long-term effect on mothers and their children. For the mother, the episode can be the precursor of chronic recurrent depression. On the other hand, for her children, it can contribute to emotional, behavioral, and cognitive problems in later life (14, 15). Since depressed mothers stop breastfeeding earlier, the infants are more likely to have episodes of diarrhea, and poor mother-infant relationships, which can affect child development and other infectious diseases (7, 16). The risk of depression increases significantly during pregnancy and clinically significant depressive symptoms are common in mid and late trimesters (17–19).

Stressful recent life events, poor social support, early life abuse, previous history of depression, abuse by an intimate partner, low levels of maternal education, low socioeconomic status, and a history of mental illness are some factors that have been associated with postpartum depression (1, 5, 11, 20, 21).

Even though different cross-sectional studies have been conducted in different parts of Ethiopia on postpartum depression, there is a dearth of research that determines causal factors for PPD using case-control studies at the community level. Therefore, this study aimed to identify the determinants of postpartum depression among mothers in Debre Tabor Town, Northcentral Ethiopia, using a community-based unmatched case-control study.

## Methods and materials

### Study design, area, and period

A community-based unmatched case-control study was conducted in Debre Tabor Town, the capital of the South Gondar Zone, which is 103 km away from Bahir Dar the capital city of Amhara National Regional State, and 666 km away from Addis Ababa capital city of Ethiopia. There are six primary schools, three health centers, and one referral hospital in the Town. According to the 2007 E.C population census report, the total population of the Zone was 2,578,906. This study was conducted from 1 to 30 December 2020.

### Source population

The source population was postnatal mothers living in Debre Tabor Town and within 6 weeks after their delivery.

#### Cases

The cases were post-partum mothers who scored ≥10 cut-off points of the EPDS and confirmed using the Diagnostic and Statistics Manual, Fourth Edition, Text Revision (22).

#### Controls

Controls were the post-partum mothers who score < 10 cut-off point of the EPDS.

### Inclusion and exclusion criteria

All postnatal mothers living in Debre Tabor Town within 6 weeks after delivery were included in this study. Postpartum mothers who had a hearing impairment or were unable to talk were not included in the study.

### Sample size and sampling procedure

To determine the sample size, an unmatched case-control study sample size determination method using Open EPI INFO version 7 software was used. Among several exposure variables, the selection of the appropriate exposure variables in controls was done based on the main interest variables of cases of postpartum depression. This research used a study done in Bahir Dar, Northwest Ethiopia, where the proportion of postpartum depression among mothers whose babies were hospitalized was 41.6% and its effect size (AOR) was 2.24 (9). During the sample size determination, we used the assumption of a 95% confidence interval, 80% power, and 1:3 case-control ratio with a 10 % non-response, yielding a total sample size of 316 (79 cases and 237 controls).

Post-natal mothers were chosen using particular household numbers. A list of households containing a coded list of post-partum mothers was available for each kebeles (the lowest administrative unit in Ethiopia containing at at-least 5,000 residents) from health extension workers at each health post. Proportional allocation to size was done to decide the study participants from each kebeles. After that, postpartum mothers were selected using a simple random sampling technique from the listed sampling frame. A house-to-house interview/screening using the Edinburg postnatal depression scale (EPDS) was undertaken based on the particular household numbers for selected postpartum mothers. Cases were identified by screening randomly selected post-partum mothers. A positive screening (score ≥10 cut-off points of EPDS) as outlined in the Diagnostic and Statistics Manual, Fourth Edition, Text Revision (DSM-IV-TR) was confirmed by a psychiatric nurse. Those confirmed postpartum mothers with PPD were taken as cases by using a consecutive sampling method until the sample size was reached. Those mothers with a negative screen test were taken as controls until the sample size was reached. The lottery method was used to select one participant if there was more than one eligible mother in the one selected household. The respondents were considered non-respondents after two revisits.

### Data collection tool and measurement

Data was collected using a structured questionnaire containing questions relating to socio-demographic characteristics, pregnancy, and birth-related factors, newborn-related factors, maternal and family-related factors, psychosocial and psychological factors, and Edinburg postnatal depression scale (EDPS). Data was collected by the data collector after informed consent and assuring the participant that their information was confidential. All the above tools were used to determine factors associated with postpartum depression among postnatal women in Debre Tabor Town.

Postpartum depression was determined when postpartum mothers scored ≥10 cut-off points of the EPDS, which has ten items with the four-Likert scale for each item and a maximum score of 30 and a minimum score of zero (23). Substance use during the current pregnancy was defined as a self-report of exposure to at least one of three substances (alcohol, khat, or tobacco) during the current pregnancy 3 months before the interview irrespective of its dose and frequency (Yes/No) (24, 25). The level of marital satisfaction was assessed using the three-item Kansas marital satisfaction scale, with each having a seven-Likert scale ranging from one (extremely dissatisfied) to seven (extremely satisfied) and a maximum score of 21 and a minimum of 3. A cut-off point of 17 and above was taken to identify satisfaction with current marital relations (26). Social support is a measure of available support that women perceive or believe that people in their network will assist in times of need and was measured by the Oslo 3-item social support scale. A score between 3 and 8 is classified as “poor support”, 9–11 is “moderate support” and 12–14 is deemed “strong support” (27). In this study, a social support score of less than nine was categorized as poor or no social support; scores ranging between 9 and 14 were considered moderate to strong support and merged as “yes” for social support.

### Study variables

#### Dependent variable

Postpartum depression

#### Independent variables

##### Women-related variables

Age, marital status, residence, religion, family income, educational status, history of family depression, history of chronic illness, and substance use (alcohol, khat, tobacco).

##### Obstetrics factors

Number of pregnancies, number of birth, number of miscarriages and stillbirths, number of live children, place of birth, use of vacuum and episiotomy, preferred sex of baby, gestational age, baby illness, history of pregnancy complication, feeding of the baby, and planned pregnancy.

##### Psycho-social factors

**Figure 1:**
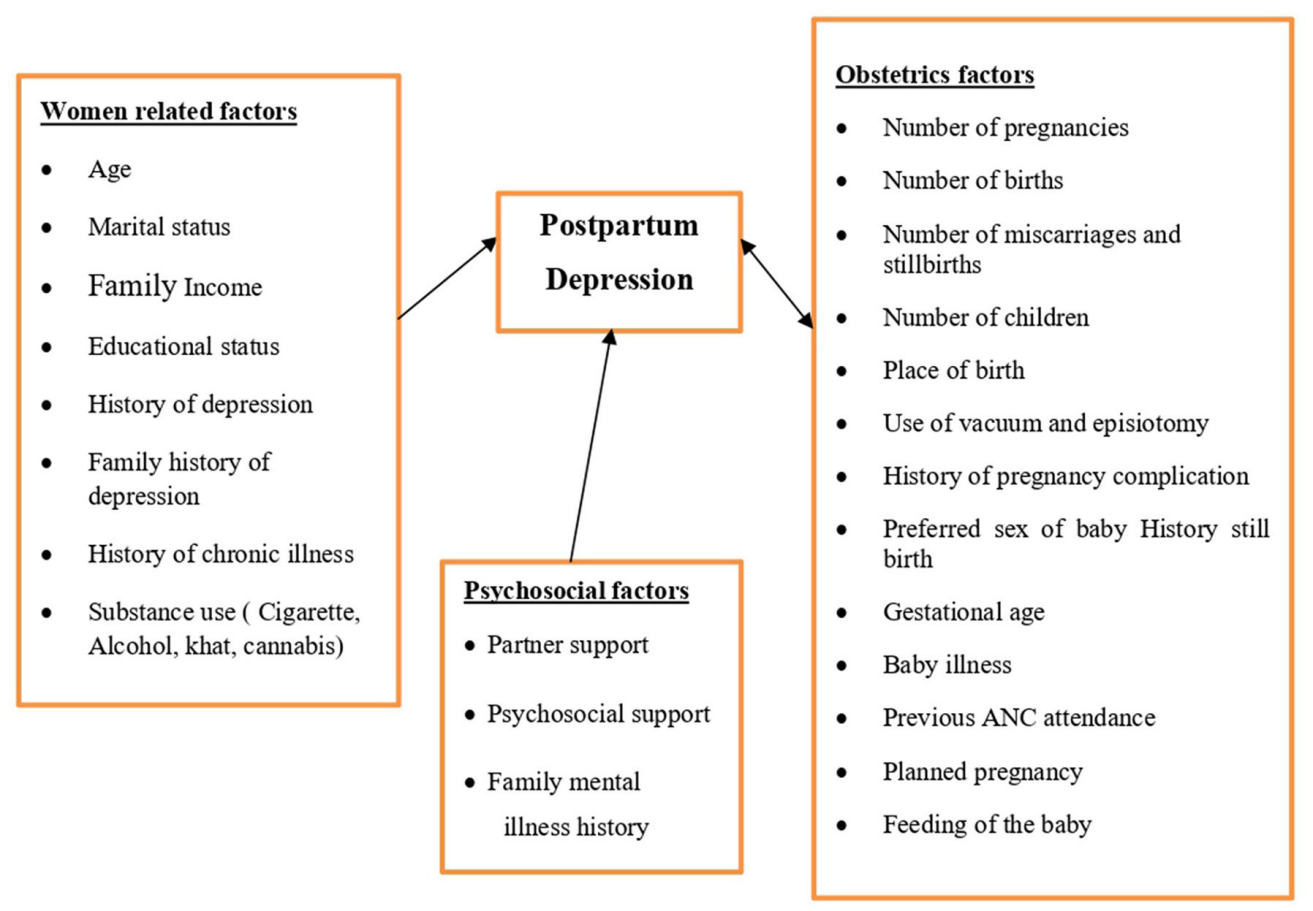
Conceptual framework of postpartum depression and associated factors.

Partner support, psycho-social support, and history of family mental illness (See Figure 1).

### Data quality management and analysis

The questionnaire was written in English, then translated into Amharic, and then back into English. The data collection instrument was pre-tested on 5% of the sample size in Woreta Town. After the questionnaire was pretested, the anticipated time required and final amendments were made. Finally, the data were entered into EpiData and the error reports were double-checked. After exporting to SPSS, cleaning was done using visualizing, sorting, and calculating frequencies data processing and analysis.

Data were cleaned, modified, and entered into EpiData version 4.6 before being imported into SPSS version 25. The frequency distribution table, graph, and summary measures were used to describe descriptive statistics. Descriptive statistics of different variables were done by cross-tabulation. Bivariate and multivariable binary logistic regression models were used for analysis. During bivariate analysis, variables with *p* < 0.25 were retained for inclusion in the multivariable analysis in the final model, then variables at *p* < 0.05 and 95% CI were considered statistically significant and independently of postpartum depression. Multi-collinearity was checked to see the linear correlation among the independent variables by using the variance inflation factor. Both Hosmer-Lemeshow's test (*P* = 0.425) and Omnibus tests (*P* = 0.000) were used to check model fitness.

### Ethical considerations

Ethical clearance was obtained from the ethical review committee of Debre Tabor University with ethical review number HSC/2222/2020. Permission to conduct the study was also obtained from the Debre Tabor Town Health office. Written consent was obtained from the respondents who were all aged above 16 years and this was confirmed by the ethical review committee. Confidentiality and anonymity were ensured throughout the execution of the study.

## Results

### Socio-demographic charcateristics of postpartum mothers

In the study, 308 mothers were included, and the response rate was 97.5%. Among the study participants, 115 (37.4%) were aged 25-34 and almost 214 (69.5%) were from rural areas. Most of the study participants 292 (94.8%) were orthodox Christians. Hundred thirty-five (43.8%) participants were illiterate and 147 (47.7%) could read and write. About 182 (59.1%) of the participants earned a monthly income of less than < 750 Ethiopian Birr (See Table 1).

**Table 1:** Sociodemographic distribution of respondents by depression category in Debre Tabor Town.

**Variables**		**Postpartum depression status**		
		**Cases (%)**	**Controls (%)**	**Total (%)**
Age	15–24	23 (26.4)	64 (73.6)	87 (28.2)
	25–34	24 (20.9)	91 (79.1)	115 (37.3)
	>35	31 (29.2)	75 (70.8)	106 (34.5)
Residence	Urban	30 (31.9)	64 (68.1)	94 (30.5)
	Rural	47 (22)	167 (78)	214 (69.5)
Educational status	Unable to read and write	41 (30.3)	94 (69.7)	135 (43.8)
	Read and write only	34 (23.1)	113 (76.9)	147 (47.8)
	Primary education	2 (15.4)	11 (84.6)	13 (4.2)
	Secondary and above	1 (7.1)	12 (92.9)	13 (4.2)
Religion	Orthodox	72 (24.7)	220 (75.3)	292 (94.8)
	Muslim	4 (40)	6 (60)	10 (3.2)
	Protestant	2 (33.3)	4 (66.7)	6 (2)
Family income	< 750	48 (26.4)	134 (73.6)	182 (59.1)
	≥750	30 (23.8)	96 (76.4)	126 (40.9)

### Obstetric and clinical characteristics of postpartum mothers

Of the 308 study participants, most respondents (194, 63%) were multigravida (give birth > 1) and 114 (37%) were primigravida (having a first child). Almost 65% of the respondents had two or more living children during the study period. About 24 (7.8%) had undergone termination of pregnancy and stillbirths, of which 14 (58%) had experienced it once and 10 (42%), two times. Nearly 298 (96.8%) of participants, delivered the last child in a health institution and 14 (4.5%) mothers were assisted with vacuum during delivery and 68 (22%) had an episiotomy. Twenty-five (8.1%) participants reported that the recent pregnancy was unplanned (See Table 2).

**Table 2:** Obstetric and clinical characteristics of respondents by depression category in Debre Tabor Town, December 2020.

**Variables**		**Postpartum depression status**		
		**Cases (%)**	**Controls (%)**	**Total (%)**
Number of pregnancies	Primigravida	26 (22.8)	88 (77.2)	114 (37)
	Multigravida	51 (26.3)	143 (73.7)	194 (63)
Number of births	Primipara	28 (23.3)	92 (76.7)	120 (39)
	Multipara	49 (26.1)	139 (73.9)	188 (61)
Number of stillbirths and miscarriage	Yes	9 (37.5)	15 (62.5)	24 (7.8)
	No	68 (24)	216 (76)	284 (92.2)
Number of live children	>1	24 (22)	85 (78)	109 (35.4)
	≥2	53 (26.6)	146 (73.4)	199 (64.6)
Place of birth	Home	5 (50)	5 (50)	10 (3.2)
	Health institution	72 (24.2)	226 (75.8)	298 (96.8)
Use of vacuum	Yes	3 (21.4)	11 (78.6)	14 (4.5)
	No	74 (25.2)	220 (74.8)	294 (94.5)
Episiotomy	Yes	17 (25)	51 (75)	68 (22.1)
	No	60 (25)	180 (75)	240 (77.9)
Recent pregnancy is planned	Yes	66 (23.3)	217 (76.7)	283 (91.9)
	No	11 (44)	14 (56)	25 (8.1)

### Newborn-related factors (recent pregnancy)

About 166 (53.9%) of the babies (from the recent pregnancy) were male and the rest were female. While 261 (84.7%) babies had a full-term gestational period, the rest were preterm. Most of the study mothers (273, 88.6%) breastfed their child, and only 34 (11%) of the newborns encountered illness during the first few months of their birth (the duration of this study) and 24 (20.8%) of mothers had a history of fetal death (See Table 3).

**Table 3:** Newborn-related factors of respondents by depression category in Debre Tabor Town.

**Variables**		**Postpartum depression status**		
		**Cases (%)**	**Controls (%)**	**Total (%)**
Preferred sex of the baby	Male	40 (29.2)	97 (70.8)	137 (44.5)
	Female	37 (21.6)	134 (78.4)	171 (55.5)
Sex of the baby	Male	38 (22.9)	128 (77.1)	166 (53.9)
	Female	39 (27.5)	103 (72.5)	142 (46.1)
Gestational age	Term	58 (22.2)	203 (77.2)	261 (84.7)
	Preterm	19 (40.4)	28 (59.6)	47 (15.3)
Current baby illness	Yes	23 (67.6)	11 (32.4)	34 (11)
	No	54 (19.7)	220 (80.3)	274 (89)
Feeding of the baby	Breastfeeding	53 (19.4)	220 (80.6)	273 (88.6)
	Formula feeding	24 (68.6)	11 (31.4)	35 (11.4)
History of fetal death	Yes	9 (37.5)	15 (63.5)	24 (20.8)
	No	68 (23.9)	216 (76.1)	284 (79.1)

### Psychosocial factors and social support among postpartum

Social support status was assessed using the Oslo-3 social support scale. Of the total study participants, the majority 298 (97%) had poor social support, 31 (10.1%) had a history of substance use, 91 (29.5%) had psychosocial stressors and 22 (7.2%) had a history of family mental illness (See Table 4).

**Table 4:** Psychosocial factors and social support among postpartum mothers by depression category in Debre Tabor Town.

**Variables**		**Postpartum depression status**		
		**Cases (%)**	**Controls (%)**	**Total (%)**
History of substance use	Yes	21	10	31 (10.1)
	No	56	221	277 (89.9)
Pyscho social stressor	Yes	34	57	91 (29.5)
	No	43	174	142 (70.5)
Family mental illness history	Yes	10	12	22 (7.2)
	No	67	219	47 (92.8)

### Determinants of postpartum depression

To identify factors independently affecting postpartum depression, all variables at a significance level of 0.25 in the bivariate analysis were retained for inclusion in the multivariable logistic analysis. From the total twelve variables that were entered in the multivariable logistic regression using the backward stepwise method, five variables were found to have a significant independent association with PPD in the final model. These were: history of substance use in the previous 3 months, current baby illness, marital satisfaction, planned current pregnancy, and breastfeeding.

The odds of developing PPD among postpartum mothers who have a history of substance use in the past 3 months were six times more likely than those who had no history of substance use (AOR: 6.47, 95% CI; 2.61, 15.74). Mothers whose babies were currently ill (first 6 months of their birth which was also the duration of our study) were three times more likely to have PPD compared to those whose babies were healthy (AOR: 3.9, 95% CI; 1.5, 10.12). Postpartum mothers with marital dissatisfaction were two times more likely to have PPD than satisfied ones (AOR: 2.41, 95% CI; 1.22, 4.75). Mothers whose current pregnancies had been unplanned were three times more likely to have PPD than those whose current pregnancies had been planned (AOR: 3.46, 95% CI; 1.32, 9.12). Breastfeeding mothers were 80% less likely to have PPD than mothers who were not breastfeeding (AOR: 0.22, 95% CI; 0.09, 0.55; See Table 5).

**Table 5:** Multivariable analysis on determinants of postpartum depression among postpartum mothers in Debre Tabor Town.

**Variables**	**Category**	**Cases**	**Controls**	**COR (95% CI)**	**AOR (95%CI)**
Residence	Urban	30	64	1.67 (0.97, 2.86)	1.25 (0.65, 2.40)
	Rural	47	167	1	1
History of substance use	Yes	21	10	8.29 (3.69, 18.59)	**6.47 (2.61, 15.74)**
	No	56	221	1	1
Pyscho social stressor	Yes	34	57	2.41 (1.41, 4.14)	1.45 (0.72, 2.92)
	No	43	174	1	**1**
Baby current illness	Yes	23	11	8.52 (3.91, 18.54)	**3.9 (1.5, 10.12)**
	No	54	220	1	1
Marital satisfaction	Not satisfied	59	128	2.64 (1.47, 4.75)	**2.41 (1.22, 4.75)**
	Satisfied	18	103	1	**1**
Planned current pregnancy	Yes	66	217	1	**1**
	No	11	14	2.58 (1.12, 5.96)	**3.46 (1.32, 9.12)**
Breast feeding	Yes	53	220	0.11(0.05, 0.24)	**0.22 (0.09, 0.55)**
	No	24	11	1	1
Gestational age	Term	58	203	0.42 (0.22, 0.81)	1.29 (0.53, 3.11)
	Preterm	19	28	1	1
Preferred sex of baby	Male	40	97	1.49 (0.89, 2.51)	1.27 (0.68, 2.37)
	Female	37	134	1	**1**
Place of birth	Home	5	5	3.14 (0.88, 11.15)	2.22 (0.43, 11.43)
	Health institution	72	226	1	1
History of fetal death	Yes	9	15	1.91 (0.8, 4.55)	1.07 (0.34, 3.41)
	No	68	216	1	1
Family mental illness history	Yes	10	12	2.72 (1.13, 6.59)	2.79 (0.96, 8.14)
	No	67	219	1	1

Bold for significant association at p < 0.05.

## Discussion

This study aimed at determining the factors associated with postpartum depression in Debre Tabor Town. Three hundred and eight mothers were included in the study with a response rate of 97.5%. The overall prevalence of postpartum depression was 77 (25%) in this study. Global studies show differing prevalence rates; for instance, 40% in Canada (11), and 34% in Jamaican (9). The prevalence rates were lower in our study compared to Canada and Jamaica. The lower prevalence rate in our study might be due to differences in residency and sample size difference. In studies carried out in the eastern Tigray zone and Mizan Aman town, the prevalence of major depression at 6 weeks postpartum was 19% (28) and 22.4% (20) respectively, which is closer to the findings from our study.

In this study, substance use had a significant association with the occurrence of PPD. This is similar to studies done in Nekemte town, West Ethiopia (29), Mizan Aman town, Southwest Ethiopia (20), and the US (30). The possible justification is that substance use has an impact on the user's emotions and behavior. In the case of substance use in postpartum mothers, there is a risk of PPD. Additionally, to deal with the feelings linked with postpartum depression, some women may turn to substance use. When life turns upside down, sleeping becomes difficult, and the stress of doing everything correctly becomes too much to bear, postpartum women turn to substances to help them cope.

The baby falling ill in the initial months had threefold odds of developing PPD compared to those who had healthier babies. This is consistent with studies done in Bahir Dar Town, Northwest Ethiopia (9) and Debre Berhan, Ethiopia (4). It is possible that the health outcome of the infants induced more stress in mothers, which eventually affected the mental wellness of the mothers.

Marital satisfaction had an inverse significant association with PPD among postpartum mothers. This is supported by studies done in Mizan Aman town, Southwest Ethiopia (20), Bandar Abbas, Iran (31), Tirana (32), Jahrom, Iran (33), and Russia (34). The reason for this could be that having a healthy relationship reduces the risk of psychological suffering. A healthy relationship can boost a mother's resilience and help her build coping skills while she adjusts to parenting. In addition to a stressful childbirth event, women may be vulnerable to PPD due to a lack of support, love, affection, and advice from their husbands. Low marital satisfaction makes it difficult to form a proper emotional bond between partners. As a result, the risk of depression increases (35).

Unplanned pregnancy was significantly associated with PPD among new mothers. This finding is similar to different studies undertaken in Ethiopia (Nekemte (29), Bahir Dar Town (9), Mizan Aman (20)), and studies done abroad (36, 37). This is because unplanned pregnancies are more likely to cause stress or worry during pregnancy, which can lead to postpartum depression. This is due to a lack of preparation for pregnancy, labor, and breastfeeding, as well as related socioeconomic compulsions. Unplanned pregnancy among partnered mothers was linked to a higher risk of psychological distress, which could lead to her partner providing less support (35).

This study found that breastfeeding mothers have fewer odds of developing PPD than non-breastfeeding mothers. This is similar to different studies (38–40). The plausible explanation might be that during successful breastfeeding, there is skin-to-skin contact, which positively promotes mother-infant bonding. Maternal-infant touch behaviors, particularly affectionate touch, are influenced by skin-to-skin contact to boost the release of oxytocin. The hormone oxytocin is produced during and after childbirth. It promotes sociability and trust among individuals while also reducing fear and anxiety (41) hence, preventing PPD. However, this result is contrary to the findings of Alder and Cox that breastfeeding mothers are at increased risk of PPD (42).

This study does not address whether or not mothers have the intention to breastfeed, and whether they face any difficulties or not. Some studies have revealed that mothers who go through negative breastfeeding experiences are at an increased risk of PPD. On the other hand, higher maternal satisfaction with breastfeeding has been associated with the absence of PPD (43). There is a very high risk of PPD among mothers who had planned to breastfeed and had not gone on to breastfeed (44). Studies show that pain and physical breastfeeding difficulty are related to postnatal depression (45), where postpartum depression may reduce rates of breastfeeding or breastfeeding may assist in a swifter recovery from the symptoms (46).

### Strength and limitation

Unlike hospital-based studies, this study used a community-based study design to determine factors associated with postpartum depression.

The limitation of the study is that it might be susceptible to social desirability bias, and to minimize potential bias we used anonymous surveys and female data collectors. This allowed respondents to answer our questions with relative ease and comfort without having to divulge any identifying details like personal names or addresses.

## Conclusion and recommendations

A significant proportion of postnatal mothers had depression, 77 (25%). Major life events and factors such as the recent history of substance abuse in the previous 3 months, current baby illness, marital satisfaction, unplanned current pregnancy, and breastfeeding were independent factors affecting the occurrence of PPD. Healthcare professionals working in maternal and child health clinics should pay special attention to postpartum mothers who have a history of substance abuse, have had an unplanned pregnancy, are non-breastfeeding mothers, and have poor marital satisfaction. Midwives and other health professionals should routinely screen new mothers for postpartum depressive symptoms and link them to mental health services, just like they would monitor other reproductive health problems in mothers attending hospitals and health centers after delivery.

## Data availability statement

The raw data supporting the conclusions of this article will be made available by the authors, without undue reservation.

## Ethics statement

The studies involving human participants were reviewed and approved by the Ethical clearance was obtained from the ethical review committee of Debre Tabor University with ethical review number HSC/2222/2020. The patients/participants provided their written informed consent to participate in this study.

## Author contributions

FD and NI conceived the original idea and were involved in proposal development, design, data collection, and analysis in all stages of the thesis. FD, WA, DA, AD, NI, YB, GY, AB, EC, and HH were involved in proposal development, analysis, and all stages of the research project. All authors read and approved the final manuscript.

## Conflict of interest

The authors declare that the research was conducted in the absence of any commercial or financial relationships that could be construed as a potential conflict of interest.

## Publisher's note

All claims expressed in this article are solely those of the authors and do not necessarily represent those of their affiliated organizations, or those of the publisher, the editors and the reviewers. Any product that may be evaluated in this article, or claim that may be made by its manufacturer, is not guaranteed or endorsed by the publisher.

## Abbreviations

DTSH: Debre Tabor Specialized Hospital
EPDS: Edinburg Postnatal Depression Scale
GYN/OBS: Gynecology and Obstetrics
ICU: Intensive Care Unit
NICU: Neonatal Intensive Care Unit
PND: Postnatal Depression
PPD: Postpartum Depression
SPSS: Statistical Package for Social Sciences.
